# Transforming health systems financing in Lower Mekong: making sure the poor are not left behind

**DOI:** 10.1093/heapol/czz098

**Published:** 2019-10-23

**Authors:** Augustine D Asante, Bart Jacobs, Virginia Wiseman

**Affiliations:** 1 School of Public Health and Community Medicine, University of New South Wales (UNSW), Samuels Building, Sydney, NSW, Australia; 2 Social Health Protection Programme, Deutsche Gesellschaft für Internationale Zusammenarbeit (GIZ), #2 Street 289, Toul Kork, Phnom Penh, Cambodia; 3 Social Health Protection Network P4H #2 Street 289, Toul Kork, Phnom Penh, Cambodia; 4 Department of Global Health and Tropical Medicine, London School of Hygiene and Tropical Medicine, 15–17 Tavistock Pl, Kings Cross, London, UK; 5 Kirby Institute, University of New South Wales (UNSW), Wallace Wurth Building, High St, Kensington, NSW, Australia

## Introduction

Equitable health financing systems are considered fundamental to the achievement of universal health coverage (UHC) and health targets under the sustainable development goals (SDGs) (Hosseinpoor *et al.*, 2018). Over the last decade, many low- and middle-income countries (LMICs) have made significant changes to their health financing systems to accelerate progress towards UHC. However, equitable financing—defined here as ensuring that the burden of paying for healthcare is in accordance with ability-to-pay and benefits from health spending are distributed on the basis of need (Tangcharoensathien *et al.*, 2015)—is far from inevitable and countries face considerable challenges in ensuring that the poor and disadvantaged are not left behind. Countries in the Lower Mekong Region (LMR) in South-East Asia provide an ideal setting for exploring such challenges, not least because of the dominant and expanding role of the private health sector and the different pace at which these countries are developing from a social and economic perspective (Cook and Pincus, 2014). This supplement shares lessons for health financing based on the experiences of three countries in the Lower Mekong Region—Cambodia, Lao PDR (Laos) and Myanmar. While each country has made a clear commitment to UHC, there are different social, political and economic circumstances that influence the path they have chosen to reach this goal. This type of context-specific evidence is critical for the design of equitable health financing systems that protect everyone, rich and poor (Tangcharoensathien *et al.*, 2011).

## Health financing in the Lower Mekong Region

Health financing in the three countries is characterized by high out-of-pocket (OOP) spending—averaging 50–60% of total health expenditure (World Bank, 2018). Vulnerability to OOP spending and poor access to healthcare in the region has many causes, including limited availability of health services to deal with emerging health conditions like chronic non-communicable diseases, ageing and disability (Meyer *et al.*, 2013; Kien *et al.*, 2016). Even where health services are available, factors such as geographical barriers, costs and acceptability by the population tend to prevent many from using services when needed (Jacobs *et al.*, 2012). In the three study countries, the vast majority of the population, especially those in the informal sector (the so-called ‘missing middle’) do not enjoy any form of financial risk protection, unlike formal sector employees who are covered by social health insurance, or poor people who receive tax-based assistance in the form of fee waivers (Bredenkamp *et al.*, 2015). Other segments of the population are covered by various social health protection schemes that often cover a limited range of health services, leaving beneficiaries still exposed to financial hardship when accessing healthcare (Shahrawat and Rao, 2012).

While health financing systems in the study countries have developed differently, shaped by country-specific circumstances, over time they have converged on similar strategies to extend access and financial protection. Table 1 provides an overview of health-financing schemes in the three study countries.

**Table 1.** Overview of health financing schemes in the three study countries

Item	Country		
	Cambodia	Lao PDR	Myanmar
Population (2016)	15.8 million	6.8 million	52.9 million
Population living below the national poverty line 2006–17 (%)	17.7	23.4	32.1
OOP payments as a % of current health expenditure	59.4	45.5	73.9
Health financing scheme	Health Equity Fund (HEF) for the poor; started in 2000 followed by nationwide expansion in 2015 Social Health insurance for formal private-sector employees; started in 2016 and for civil servants since 2018	State Authority for Social Security (SASS) for civil servants; started in 1995 Social security organization (SSO) for private employees; started in 2001 Community-Based Health Insurance (CBHI) for non-poor people in the informal sector; started in 2002 HEF for the poor; started in 2004.	Social health insurance scheme or Social Security Scheme (SSS) for state enterprise employees, civil servants and employees of public and private firms with five or more employees; started in 1956.
Benefit package	Outpatient and inpatient services with food stipends and transport reimbursement for hospitalized patients under HEF	Outpatient and inpatient services, except HEF that also covers travel and food costs for inpatients.	Outpatient and inpatient plus medicine, laboratory and transportation in case of referral outside urban areas.
Scheme coverage	About 28% of the total population was covered by these schemes in 2018 (i.e. 4 million people).	About 27.2% of the population was covered by these schemes in 2014.	About 3% of the eligible population was covered by the scheme in 2014.

OOP, out-of-pocket.

*Sources:* World Health Organization (2018); World Bank (2018); Myint *et al.* (2018); and Sydavong *et al.* (2019).

## Content of this supplement

All four papers in this supplement evaluate the degree of equity in health financing, albeit from different angles. Two papers (Asante *et al.* and Nagpal *et al.*) explore the benefit incidence of health financing. Asante *et al.* focus on the distribution of healthcare benefits across different socioeconomic groups in Cambodia. They report, among other things, that the benefits from health spending in the public sector in Cambodia are generally distributed in favour of the poor, reflecting the level of need for health services. The authors also note that over 50% of total health expenditure and healthcare delivery remains with the private sector which distributes healthcare benefits in favour of the rich. Given the significant proportion of poor Cambodians who use private providers, it will be difficult for Cambodia to achieve UHC if this challenge is not comprehensively addressed.

Nagpal et al. examine the effect of a free universal maternal and child health scheme implemented by the Laos government on equity of access to health services and financial protection. Financial protection is measured in terms of the ability to access free healthcare at the point of delivery—with special emphasis on ethnic minority women. Evidence from this article points to persistent and large inequities in access and financial protection that cannot be ignored. Significant differences were also observed in the utilization of health services by economic status and ethnicity. These inequities are accentuated by issues related to the distribution and nature of human resources, supply-side readiness and quality of care provided across different geographical areas.

The remaining two papers examine the effects of paying for healthcare. The paper by Por *et al.* takes a closer look at the effects of OOP spending at the individual level in Cambodia, using distress financing (borrowing with interest to pay for healthcare) as an indicator of financial hardship. Their findings suggest that a large proportion of Cambodian households experience distress financing and a key determinant of this is household poverty, even for households covered by the HEF. Finally, Ergo and colleagues explore the consequences of relying excessively on OOP spending as the main source of health financing. The authors use the most recent nationally representative survey, the 2015 Myanmar Poverty and Living Conditions Survey, that also includes the first data on health expenditure in conflict-affected areas. This article indicates that a substantial number of households in Myanmar, many of whom are already living below the national poverty line, experience catastrophic and further impoverishing health care payments. The coping mechanisms adopted by these households include borrowing and selling of household assets while a substantial proportion do not seek care at all as a cost-saving measure.

## Lessons emerging from this supplement

Together, these papers demonstrate that while progress has been made, there is still much to be done if UHC is to become a reality in the three LMR countries, not least because of limited financial risk protection; high utilization of private health facilities; and the borrowing and selling of household assets to cope with the high OOP expenditure.

There is little doubt that governments in the LMR are making efforts to protect their citizens against impoverishing healthcare spending. All of them have committed to UHC and are restructuring their health financing systems to expand coverage along with financial protection. However, the level of protection currently offered to the poor and other vulnerable groups is insufficient to achieve the goal of UHC. All three country case-studies show that a large proportion of the population still incurring high OOP expenditure when accessing healthcare. Existing social protection schemes do not appear to be comprehensive enough, partly because of limited domestic funding for these schemes. Despite rapid economic growth, countries in the LMR face numerous economic challenges that affect their ability to mobilize domestic revenue. With national budgets stretched to the limit, these countries and many other LMICs, are unable to allocate sufficient funds to expand social assistance interventions (OECD, 2013).

In the absence of a sufficiently funded, carefully designed and implemented public health system, the private sector is shown to flourish in the LMR. In both Cambodia and Myanmar, private providers operate in a minimally regulated environment but are deemed more responsive to patient needs and demands than the public sector, accounting for a large proportion of service utilization. Since most OOP spending occurs in this sector, it is paramount that strategies are developed to regulate and monitor their activities, especially in the areas of service quality and fee-setting. The high OOP expenditure associated with the private sector often result from poor quality of treatment or supplier-induced demand that exposes users to unnecessary costs (Hanson *et al.*, 2008; Morgan *et al.*, 2016). Although there is no ‘magic bullet’ in terms of how best to engage the private sector in a resource-constrained environment, a strong public system appears to enable improved service delivery by the private sector (Morgan *et al.*, 2016; Clarke *et al.*, 2018; Binagwaho and Ghebreyesus, 2019).

A key consequence of the high OOP expenditure and limited financial risk protection in the three countries is the harmful practice of borrowing and selling household assets. This borrowing is encouraged by the easy access to finance from private financial institutions. For example, in Cambodia, loans are easily accessible from numerous banks and microfinance institutions and it is not uncommon for people to borrow just to pay the interest on outstanding loans (Bylander *et al.*, 2019). Papers in this special supplement highlight the need for improved regulation of the private finance industry including closer monitoring of the terms and conditions for loans from these institutions, especially the long loan repayment periods. Many respondents in the study by Por and colleagues had not paid off the loans they had taken out a year or more prior to the study. In Myanmar, as Ergo *et al.* observe, foregoing health care altogether because of financial barriers was a common coping strategy, especially by those unable to access finance.

As countries focus on increasing coverage, it is paramount that the poor and vulnerable—who are often the most difficult to reach—are not left behind. This supplement reminds us that without adequate focus on equity, it may be some time before the benefits of UHC trickle down to everyone.
